# Whole-body TSPO-PET reveals stage-dependent disruption of systemic immune organ interactions in glioblastoma

**DOI:** 10.1007/s00259-026-08057-0

**Published:** 2026-07-14

**Authors:** Gloria Mueller, Artem Zatcepin, Laura M. Bartos, Leonie Hoermann, Lena Wesser, Ha Eun Park, Justus F. Thevis, Marlies Haertel, Lea H. Kunze, Giovanna Palumbo, Zeynep Ilgin Kolabas, Simon Lindner, Rudolf A. Werner, Nathalie L. Albert, Louisa von Baumgarten, Sibylle Ziegler, Matthias Brendel

**Affiliations:** 1 Department of Nuclear Medicine, LMU University Hospital, LMU Medizin, Ludwig-Maximilians-Universität München, Munich, Germany; 2 Department of Nuclear Medicine, Hannover Medical School (MHH), Hannover, Germany; 3 Institute of Clinical Neuroimmunology, LMU University Hospital, LMU Medizin, Ludwig-Maximilians-Universität München, Munich, Germany; 4 Biomedical Center (BMC), Faculty of Medicine, LMU Munich, Martinsried, Germany; 5 Russell H. Morgan Department of Radiology and Radiological Sciences, Johns Hopkins School of Medicine, Baltimore, MD USA; 6 German Cancer Consortium (DKTK), Partner Site Munich, A Partnership Between German Cancer Research Center (DKFZ) and LMU Munich, Munich, Germany; 7 Bavarian Cancer Research Center (BZKF), Partner Site Munich, Munich, Germany; 8 Department of Neurology, LMU University Hospital, LMU Medizin, Ludwig-Maximilians-Universität München, Munich, Germany; 9 Department of Neurosurgery, LMU University Hospital, LMU Medizin, Ludwig-Maximilians-Universität München, Munich, Germany; 10 German Center for Neurodegenerative Diseases (DZNE), Munich, Germany; 11 Munich Cluster for Systems Neurology (SyNergy), Munich, Germany

**Keywords:** TSPO-PET, Whole-body, Mouse, Glioblastoma, Inflammation, Immune system

## Abstract

**Purpose:**

Strong expression of the 18 kDa translocator protein (TSPO) is observed in glioma and tumor-associated myeloid cells within the tumor microenvironment. However, TSPO expression also extends beyond the tumor and may reflect systemic immune regulation. We therefore assessed inter-organ associations of TSPO expression in mice at different stages of glioblastoma using whole-body TSPO-PET imaging.

**Methods:**

Whole-body TSPO-PET images were acquired using [^18^F]GE-180 in sham-inoculated mice (*n* = 18) and glioblastoma-bearing mice at early (5–13 days, *n* = 20) and late (18–19 days, *n* = 29) stages. The tumor and organs (brain, heart, lungs, skull, and various bones) were segmented to compare TSPO-PET signals between mouse cohorts. Pearson correlation served to analyze cohort-specific organ-organ interaction, and deviations were quantified using correlation abnormality scores. [^18^F]DPA-714 TSPO-PET scans (*n* = 53) of a validation cohort served for longitudinal assessment of TSPO organ-organ interaction, which was analyzed relative to tumor stage and survival.

**Results:**

Late-stage glioblastoma mice showed increased TSPO expression in the tumor region, but no significant TSPO alterations in other organs. In contrast, organ-organ interactions, including lungs and non-tumor brain regions, were disrupted in early- and late-stage glioblastoma mice compared to sham mice. Late-stage glioblastoma mice had high correlation abnormality scores across several organ systems, as was also observed in data obtained with the second TSPO tracer. Longitudinal TSPO organ-organ interactions, but not TSPO-PET signals in single organs, were associated with tumor stage and survival.

**Conclusions:**

Glioblastoma induces stage-dependent systemic TSPO alterations and organ–organ interaction changes, suggesting that whole-body TSPO-PET network analysis may track tumor-associated immune dynamics.

**Supplementary Information:**

The online version contains supplementary material available at https://doi.org/10.1007/s00259-026-08057-0.

## Introduction

Positron emission tomography (PET) imaging of immune cell targets and, in particular, the 18 kDa translocator protein (TSPO) is increasingly used in preclinical research on neuroinflammatory and neurodegenerative diseases [1–4]. TSPO-PET imaging has already been established for the prognosis and detection of tumor heterogeneity in gliomas [5, 6]. It could serve as a potential imaging biomarker to characterize the immunosuppressive phenotype of glioblastoma during immunomodulatory treatment, despite its expression by different cell types, including glioma cells, activated microglia, and infiltrating immune cells [7]. Beyond neuroinflammatory conditions, Thackeray et al. [8] demonstrated evidence of concomitant inflammation in the heart and the brain of mice and patients with ischemic heart disease using TSPO-PET. Moreover, the TSPO-PET tracer [^18^F]DPA-714 has been used to measure organ-level immunoreactivity and the resulting dysfunction in systemic inflammatory response syndrome and sepsis [9]. These studies revealed that TSPO is not only expressed in the brain but also in the bone marrow and various peripheral tissues, such as the heart, colon, liver, and lungs [9].

The present study aimed to investigate correlations between TSPO expression in the glioblastoma lesion and other brain regions, as well as in various organs and bone compartments, to gain insights into tumor-associated systemic immune processes. Additional analyses were performed with [^18^F]DPA-714 TSPO-PET scans of glioblastoma-bearing mice to cross-validate [^18^F]GE-180 TSPO-PET signals and for longitudinal assessment of TSPO organ-organ interaction relative to tumor stage and survival.

## Materials and methods

### Animals

All animal experiments followed an established standardized protocol [3]. Briefly, female, 6–8-week-old C57BL/6 mice were randomly divided into glioblastoma and sham-injected mice and scanned with [^18^F]GE-180 (*n* = 67) or [^18^F]DPA-714 (*n* = 39).

Within the [^18^F]GE-180 group, 49 mice were subject to stereotactic inoculation with 100,000 SB28-eGFP cells (glioblastoma-bearing mice), and 18 mice were inoculated with 2 µL of saline (sham-injected mice).

Within the [^18^F]DPA-714 group, 27 mice were subject to stereotactic inoculation with 1,000 SB28-eGFP cells (glioblastoma-bearing mice), and 12 mice were inoculated with 2 µL of Hanks Balanced Salt Solution (HBSS) (sham-injected mice).

### Small-animal PET/CT imaging

Small-animal PET procedures were pre-established, using a standardized protocol for acquisition and post-processing [4, 10]. All images were acquired using the Mediso nanoScan PET/CT system (Mediso Ltd, Budapest, Hungary). For a schematic overview of the mouse cohorts and the PET/CT scans performed, see Supplementary Fig. S1.

[^18^F]GE-180 PET recordings (12.6 ± 3.3 MBq, *n* = 67) with an emission window of 40–60 min after injection were obtained to measure TSPO expression. PET imaging was carried out 5–25 days post-inoculation. Glioblastoma-bearing mice underwent [^18^F]GE-180 PET at an early (5–13 days post-inoculation, *n* = 20) or a late time point (18–19 days post-inoculation, *n* = 29), while sham-injected mice received [^18^F]GE-180 PET after 6–25 days post-inoculation (*n* = 18).

[^18^F]DPA-714 PET recordings (15.83 ± 3.84 MBq, *n* = 71) with an emission window of 40–60 min after injection were obtained to measure TSPO expression. PET imaging was carried out 11–32 days post-inoculation. Glioblastoma-bearing mice underwent [^18^F]DPA-714 PET imaging at early (11–15 days, *n* = 13), intermediate (17–21 days, *n* = 23), and late (24–32 days, *n* = 17) time points after inoculation, while sham-injected mice received [^18^F]DPA-714 PET after 14–21 days post-inoculation (*n* = 18). To monitor long-term tumor progression, 1–3 PET/CT scans were acquired per glioblastoma-bearing mouse, depending on tracer availability and the survival of the mice (total number of mice scanned once: *n* = 8; total number of mice scanned twice: *n* = 12; total number of mice scanned three times: *n* = 7).

### Image preprocessing and image analysis

#### Spatial co-registration

PET and CT data were co-registered using the PMOD Fusion Tool (version 3.5, PMOD Technologies LLC, Zurich, Switzerland). Additionally, for spatial co-registration of the brain, the PET and CT data were cropped to the head region and fused. A study-specific CT template (i.e., to consider minor anatomical deviation due to inoculation) was generated [11, 12] by averaging the cropped CT scans (*n* = 12) rigidly matched to a T2w-MR template in the Ma-Benveniste-Mirrione space [11, 12]. All individual CT scans were subsequently deformably registered to the respective CT template using the SPM5 procedure. The resulting transformations were then applied to the PET scans. Finally, the PET scans were smoothed using a Gaussian filter with 1 mm full-width-at-half-maximum to reduce noise.

#### Assessment of TSPO-PET tracer uptake

A study-specific atlas comprising a cerebellum, a contralateral hemisphere, and a skull volume of interest (VOI) was created in the Ma-Benveniste-Mirrione space [11, 12] using PMOD View Tool (version 3.5, PMOD Technologies LLC, Zurich, Switzerland). The cerebellum VOI was derived from the Ma-Benveniste-Mirrione atlas [11, 12] using intensity-based threshold segmentation. The contralateral hemisphere VOI was manually delineated on the right hemisphere based on anatomical landmarks, and the skull VOI was extracted from the study-specific CT template using intensity-based threshold segmentation within a predefined oval-shaped volume centered on the upper right calvarium, followed by morphological closing. The mean TSPO-PET tracer uptake in each VOI (cerebellum, contralateral hemisphere, and skull) was automatically extracted from all PET scans using a custom Python script [13].

The tumor region, heart, lungs, bones (humerus, vertebra, pelvis, and femur), and the whole body VOI were segmented using PMOD View Tool. The heart and the tumor region were segmented using a threshold method adapted from an earlier PET study [14]. The mean tracer uptake of each VOI was derived from automatically generated 3D VOIs obtained by PMOD isocontour detection within predefined search volumes.

For the heart and tumor, the hot search mode was applied with a threshold of 70% of the intensity range (max–min) within predefined volumes: an oval-shaped volume (*V* = 70 mm^3^) centered around the myocardium and a spherical volume (*r* = 1.5 mm) centered on the tumor region in glioblastoma-bearing mice or the injection site in sham-injected mice.

The mean tracer uptakes in bones were derived using the hot search mode with a threshold of 30–35% of the intensity range (max–min) within predefined CT-based volumes centered around the respective bones. The mean tracer uptake in lungs was determined using the cold search mode with a threshold of 15–20% of the intensity range (max–min) within a predefined CT-based volume centered around the lungs.

The mean whole-body tracer uptake was obtained using hot search mode with a threshold of 2% of the intensity range (max–min) within a predefined volume including the whole mouse.

The mean tracer uptake (activity concentration) in each VOI was normalized to the mean whole-body activity concentration of each mouse, thereby yielding a unitless VOI-to-whole-body tracer uptake ratio.

VOI placement was confirmed by visual inspection in axial, sagittal, and coronal projections to minimize spill-over effects from adjacent tissue.

### Assessment of TSPO-PET interregional correlation coefficients (ICCs) and correlation abnormality score (CAS)

Interregional correlation coefficient (ICC) assessment of TSPO-PET was performed according to an established protocol [1, 15]. Briefly, Pearson’s correlation coefficients were calculated for all VOI pairs, and bootstrapping with replacement was performed (10,000 iterations, each matching the original sample size). Pearson’s *R* values were subsequently normalized via Fisher’s *R*-to-*Z* transformation [16], and the mean *Z* across all resampled datasets was defined as the ICC. As Pearson’s correlation coefficients were calculated for all VOI pairs simultaneously, Benjamini–Hochberg false-discovery rate (FDR) [17] adjustment was applied to the resulting *p*-values to control for multiple comparisons, where *p*_FDR_ < 0.05 was used as a significance threshold.

To assess deviations in glioblastoma-bearing mice relative to the sham cohort, we introduced a quantitative score termed the *correlation abnormality score (CAS)* at two levels: the *individual CAS*, quantifying the deviation of a single VOI in a single mouse from the normal correlation structure, and the *cohort CAS*, quantifying the mean deviation of a VOI pair across all mice in a given cohort from the normal correlation structure.

The CASs were calculated as follows:

1. For all mice of the reference (sham) cohort, mean tracer uptake ratios were extracted for all VOIs.
2. Using these values, a linear fit was calculated for each pair of VOIs, representing the ICC.
3. For each mouse to be assessed, mean tracer uptake ratios were extracted for the same set of VOIs.
4. For each VOI pair, the perpendicular distance *D* from the mouse’s pair of tracer uptake ratios to the corresponding linear fit calculated in step 2 was calculated.
5. Based on these distances, the CASs were defined as follows:

The *individual CAS* of VOI *i* and mouse *k* was defined as the sum of the perpendicular distances across all VOI pairs involving VOI *i*:

$$\:CA{S}_{\:i}^{k}=\:{\sum\:}_{j=1,\:j\ne\:i}^{N=10}{D}_{i,j}$$

The *cohort CAS* for a VOI pair *i*,* j* was defined as the mean perpendicular distance across all mice *k* within a cohort *c*:

$$\:CAS_{i,j}^c=\:\frac1M{\sum\:}_{k=1}^MD_{i,j,k}$$

where *N* denotes the total number of VOIs and *M* the number of mice in the respective cohort.

To calculate the CASs in the reference cohort itself, a leave-one-out approach was applied.

The CASs were validated (Supplementary Fig. S2) and calculated for the [^18^F]GE-180 cohorts (late-stage: *n* = 29, early-stage: *n* = 20, sham: *n* = 18) and for the [^18^F]DPA-714 cohorts (late-stage: *n* = 17, intermediate-stage: *n* = 23, early-stage: *n* = 13, sham (reference): *n* = 18).

### Statistical analysis

Prior to group comparisons, the Shapiro-Wilk normality test [18] was applied to all values, confirming non-normal distributions for tracer uptake ratios, ICCs, and CAS values across all cohorts. Accordingly, non-parametric tests were used for all subsequent statistical comparisons.

To test for differences in the tracer uptake ratios or individual CAS values between cohorts, statistical significance was determined using the Mann-Whitney U-test [19]. The tracer uptake ratios are reported as the percentage difference in the median tracer uptake ratios between glioblastoma-bearing mice and sham-injected mice. The corresponding individual CAS values are reported as the median individual CAS. As Mann-Whitney U-tests were performed simultaneously across all VOIs and cohort comparisons, Benjamini–Hochberg FDR adjustment was applied to *p*-values, where *p*_FDR_ < 0.05 was used as a significance threshold.

The Wilcoxon signed-rank test (two-tailed) was used to compare median ICCs and cohort CAS values between cohorts on a VOI-pair-matched basis. The corresponding results are reported as Q2 (Q1, Q3), where Q2 is the median ICC or median cohort CAS, Q1 is the first quartile, and Q3 is the third quartile. *p* < 0.05 was used as a significance threshold.

The above-mentioned calculations and statistical tests (summarized in Supplementary Table S1) were performed using a custom-made Python script (Numpy 2.0.1, Pandas 2.2.2, Scipy 1.14.0, Pingouin 0.5.4 libraries, Python 3.12) [13].

### Longitudinal progression

To facilitate testing for longitudinal associations of TSPO expression, we changed the amount of inoculated SB28 cells from 100,000 to 1,000 to prolong the possible observation period of SB28 mice [20]. The [^18^F]DPA-714 TSPO-PET recordings of mice scanned at least twice (*n* = 45, mice scanned twice: *n* = 12; mice scanned three times: *n* = 7) were used for long-term analysis of the tumor progression.

To model the temporal trajectory and the time before death of the individual [^18^F]DPA-714 TSPO-PET CASs, generalized additive mixed models (GAMMs) were fitted. Longitudinal individual CASs were plotted against post-inoculation scan time and time before death. Individual mouse trajectories were shown as grey lines, with individual CAS values shown by points. The significance of smooth terms was assessed using *p*-values and interpreted together with effective degrees of freedom (EDF). The model-estimated temporal effect was overlaid as a black line, with a shaded ribbon indicating the 95% confidence interval (CI).

## Results

### Changing TSPO-PET signals of organs in glioblastoma-bearing mice compared to sham-injected mice

**Fig. 1:**
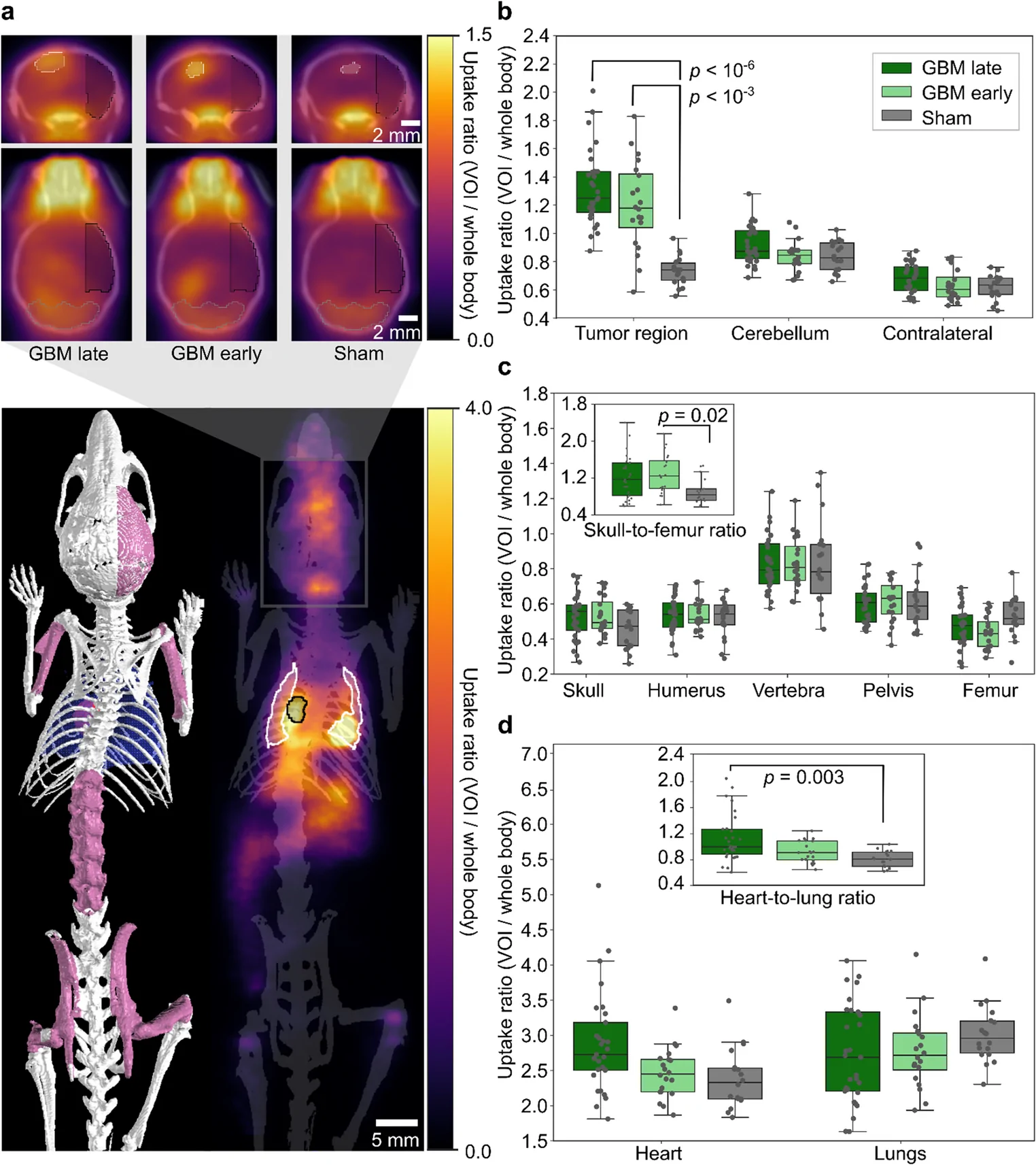
Mean [^18^F]GE-180 uptake ratios of various volumes of interest (VOIs).** a** Fused average PET and CT scans of late-stage (GBM late) and early-stage (GBM early) glioblastoma-bearing mice and sham-injected mice (sham), showing the tumor region, cerebellum, and contralateral hemisphere outlined in white, black, and grey, respectively (top). Bottom left, the 3D-rendered skeleton of a late-stage glioblastoma-bearing mouse with VOIs in red (heart), blue (lungs), and pink (bones); bottom right, the corresponding fused TSPO-PET scan with the heart and lungs outlined in black and white, respectively. Mean uptake ratios in the** b** tumor region, cerebellum, and contralateral hemisphere, **c** bones, and **d** heart and lungs of late-stage and early-stage glioblastoma-bearing mice and sham-injected mice

First, to identify tumor-associated immune processes, we investigated [^18^F]GE-180 TSPO-PET signals in different VOIs of the mouse body (Fig. 1a).

In mice with glioblastoma, TSPO-PET signal was significantly increased in the tumor region at both early (+ 59%, *p* = 0.0003) and late stages (+ 69%, *p* < 10^− 6^) compared to sham-injected mice (Fig. 1b). In the cerebellum and the contralateral hemisphere, TSPO-PET signal was similar in early-stage glioblastoma-bearing mice compared to sham-injected mice (cerebellum: +2%, *p* = 0.96; contralateral: -5%, *p* = 0.96) and tended to increase in late-stage glioblastoma-bearing mice compared to sham-injected mice (cerebellum, + 6%, *p* = 0.20; contralateral hemisphere: +8%, *p* = 0.21), indicating whole brain inflammation in the late tumor stage (Fig. 1b).

In bones (Fig. 1c), TSPO-PET signal showed a trend toward increased expression in the skull when comparing late-stage glioblastoma-bearing mice against sham-injected mice (+ 10%, *p* = 0.20 (late-stage), + 1%, *p* = 0.25 (early-stage)). Similar TSPO-PET signal was observed between glioblastoma-bearing mice and sham-injected mice in vertebrae and pelvis (+ 1 to + 7%, *p* = 0.96). In contrast, the femur (-8 to -16%, *p =* 0.06–0.22) and the humerus (-0.2 to -6%, *p =* 0.96) tended towards lower levels of TSPO-PET signal in glioblastoma-bearing mice compared to sham-injected mice, leading to a significant difference in the TSPO-PET signal for the skull-to-femur-ratio in early-stage glioblastoma-bearing mice compared to sham-injected mice (+ 42%, *p* = 0.02).

TSPO-PET signal in the heart showed a trend toward increased expression (+ 17%, *p* = 0.05 (late-stage), + 5%, *p* = 0.64 (early-stage)). In comparison, TSPO-PET signal of the lungs tended to be decreased (-9%, *p* = 0.24 (late-stage), -8%, *p* = 0.21 (early-stage)) when comparing glioblastoma-bearing mice against sham-injected mice (Fig. 1d), leading to a large difference in the [^18^F]GE-180 uptake ratio for these organs (heart-to-lungs ratio) in late-stage glioblastoma-bearing mice compared to sham-injected mice (+ 23%, *p* = 0.003) (Fig. 1d).

### Whole-body PET reveals altered organ-organ interaction of TSPO expression in glioblastoma-bearing mice

As TSPO-PET has not yet been used to analyze interactions between different immune cell compartments in various organs, we calculated pairwise Pearson correlations between the TSPO-PET signals of individual organs to analyze group-specific connectivity patterns. In glioblastoma-bearing mice, the correlation between the tumor region and the contralateral hemisphere was strong (late: *R* = 0.77, *p* < 10^− 4^; early: *R* = 0.78, *p* = 0.0009; sham: *R* = 0.42, *p* = 0.25), as expected from previous findings of TSPO-positive immune cell activation in the contralateral hemisphere [21]. Similarly, in glioblastoma-bearing mice, the correlation between the tumor and the cerebellum (late: *R* = 0.71, *p* = 0.0003; early: *R* = 0.74, *p* = 0.002) as well as the tumor and the skull (late: *R* = 0.63, *p* = 0.002; early: *R* = 0.65, *p* = 0.02) was stronger than in sham-injected mice (*R* = 0.26–0.30, *p* = 0.40–0.53).

**Fig. 2:**
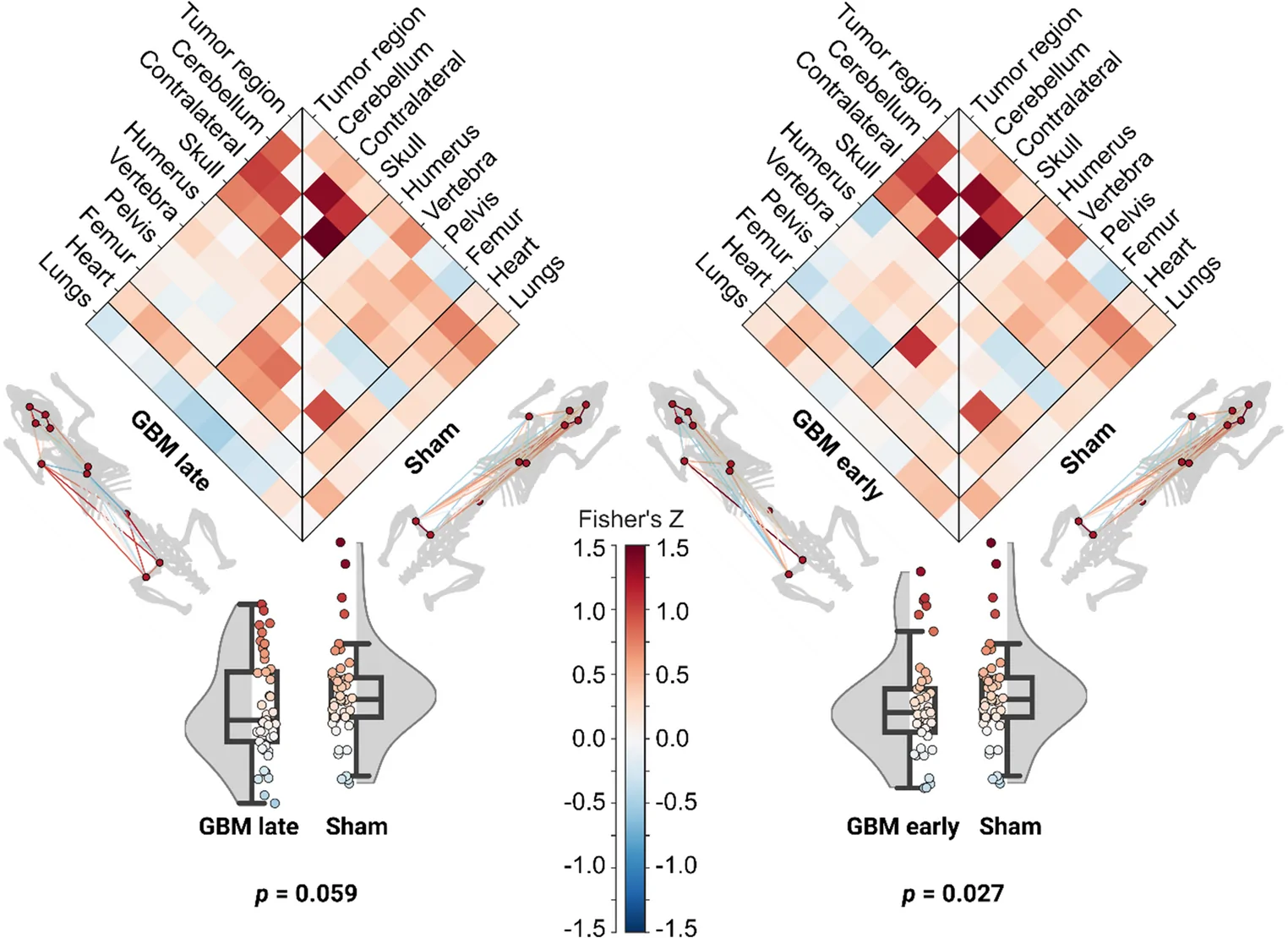
Assessment of interregional correlation coefficients (ICCs) of [^18^F]GE-180 TSPO-PET signal in late-stage (GBM late) and early-stage glioblastoma-bearing (GBM early) mice versus sham-injected mice (sham). Plots show ICC heat maps comparing the correlations between various organs in late-stage glioblastoma-bearing mice with sham-injected mice (left), and early-stage glioblastoma-bearing mice with sham-injected mice (right)

Overall, early-stage glioblastoma-bearing mice exhibited decreased TSPO organ-organ correlations compared to sham-injected mice (median ICC: early-stage 0.20 (0.05, 0.38) and sham 0.30 (0.16, 0.47), *p* = 0.027). Decreased TSPO organ-organ correlations were also observed in late-stage glioblastoma-bearing mice compared to sham-injected mice, primarily driven by negative correlations of the lungs with other VOIs (median ICC: late-stage − 0.26 (-0.32, -0.13) and sham 0.25 (0.16, 0.46), *p* = 0.004), and weaker correlations of the cerebellum with other VOIs (median ICC: late-stage 0.14 (0.01, 0.70) and sham 0.58 (0.41, 0.73), *p* = 0.098); however, the overall difference did not reach significance (median ICC: late-stage 0.14 (-0.03, 0.51) and sham 0.30 (0.16, 0.47), *p* = 0.059) (Fig. 2). Overall, there were fewer strong positive and more negative correlations (i.e., lung connections) in glioblastoma-bearing mice than in sham-injected mice (Fig. 2).

### Correlation abnormality score (CAS) for assessment of individual alterations in organ-organ interaction in glioblastoma-bearing mice

To quantify the deviation of TSPO organ-organ interaction in glioblastoma-bearing mice and compare it to sham-injected mice, the individual CAS and the cohort CAS were introduced. The individual CAS is a score per mouse that expresses the deviation of a specific region from the norm in the overall network of regions. The cohort CAS quantifies the deviation of regional correlations from the reference cohort.

**Fig. 3:**
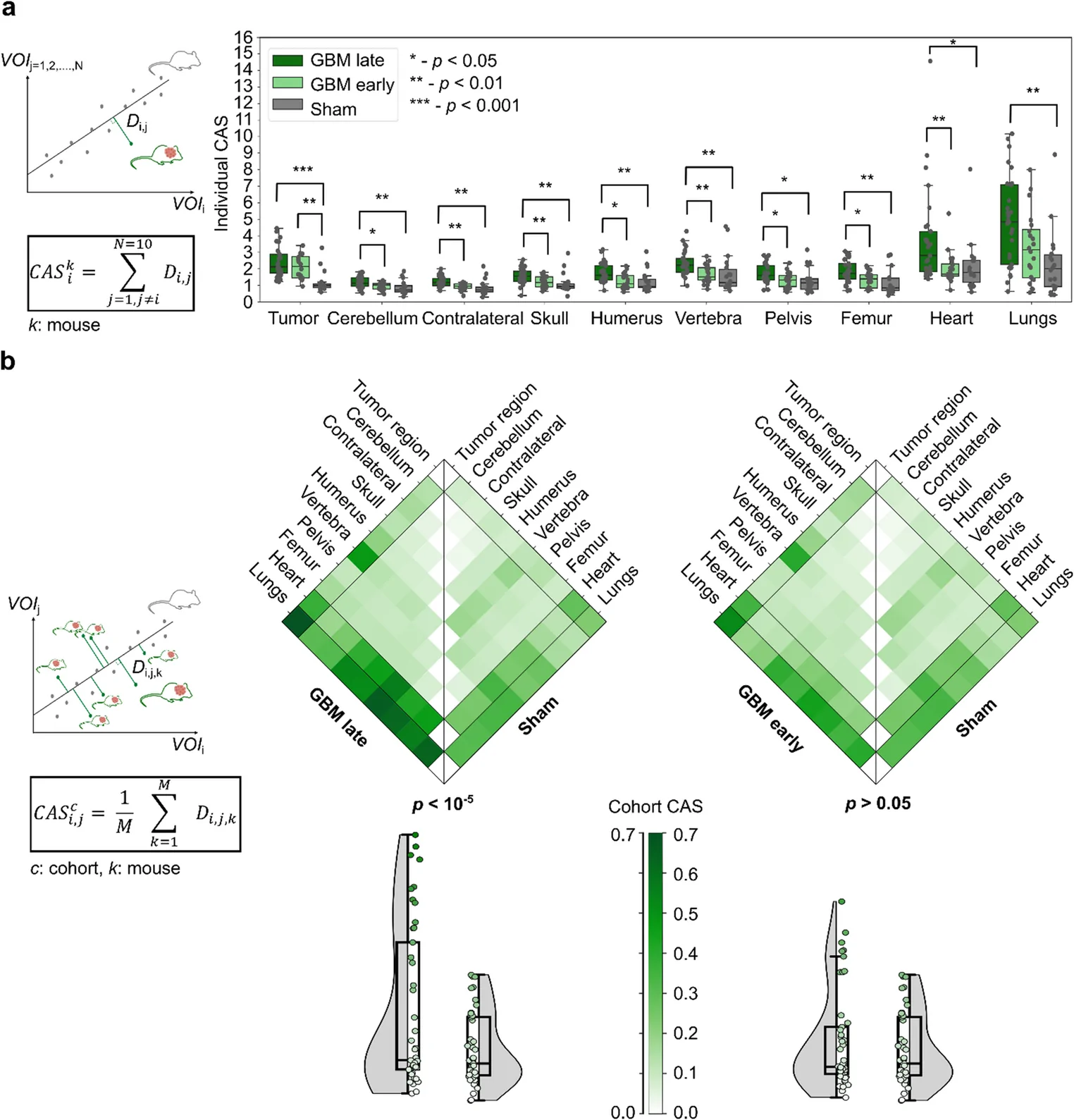
Assessment of correlation abnormality scores (CAS) showing the deviation of organ-organ correlations at the individual and cohort level of late-stage (GBM late) and early-stage glioblastoma-bearing mice (GBM early) in comparison with sham-injected mice (sham) using [^18^F]GE-180 TSPO-PET signals. **a** Individual CAS for all volumes of interest (VOIs) and **b** cohort CAS of all VOI pairs

In early-stage glioblastoma-bearing mice, a significant change in the median individual CAS was observed only in the tumor region (2.1) compared to sham-injected mice (1.0, *p* = 0.001) (Fig. 3a). In contrast, significant differences in the median individual CAS between late-stage glioblastoma-bearing mice and sham-injected mice were observed in all regions, with the tumor region (2.1 versus 1.0, in sham-injected mice, *p* < 10^− 4^) comprising the most abnormal organ-organ interactions (Fig. 3a).

In sham mice, the heart and lungs exhibited higher median individual CAS (1.8 and 2.0, respectively) than the rest of the organs (< 1.2) (Fig. 3a), indicating the TSPO-PET signals in the heart and lungs vary more than other regions in physiological conditions. Compared to sham-injected mice, the median individual CAS value for the heart remained constant, while that for the lungs increased in glioblastoma-bearing mice in the early stage (heart: 1.7, *p* = 0.85; lungs: 3.1, *p* = 0.14). Median individual CAS values for the heart and lungs reached their maximum value in the late stage (heart: 2.8, *p* = 0.02; lungs: 4.8, *p* = 0.005).

In the late-stage glioblastoma mice, the median cohort CAS over all organ pairs was significantly higher compared to sham-injected mice (0.13 (0.11, 0.42) versus 0.12 (0.09, 0.24) in sham-injected mice, *p* < 10^− 5^), while the median cohort CAS of the early-stage glioblastoma-bearing mice did not differ from sham-injected mice (0.11 (0.1, 0.21), *p* = 0.14) (Fig. 3b).

The most substantial impact on the elevated median cohort CAS over all region pairs in the late-stage glioblastoma cohort was attributed to the median cohort CAS of the lungs region pairs (0.56 (0.52, 0.64)), heart region pairs (0.42 (0.37, 0.46)) and tumor region pairs (0.16 (0.14, 0.37)) which increased significantly compared to those observed in sham-injected mice (lung: 0.27 (0.23, 0.31), *p* = 0.004, heart: 0.25 (0.24, 0.28), *p* = 0.004, tumor: 0.10 (0.09, 0.13), *p* = 0.008) (Fig. 3b).

### Longitudinal assessment of organ-organ interactions

Time points of PET imaging were set to achieve similar tumor sizes at early-stage and late-stage in both cohorts, with the opportunity for an additional intermediate time point. As expected, the average survival time in mice with glioblastoma was longer after inoculation of 1,000 cells compared to 100,000 cells (28.9 vs. 21.3 days; *p* < 0.001). The glioblastoma cohort with 1,000 inoculated cells (and the associated sham cohort) was measured using the tracer [^18^F]DPA-714 (early-stage: day 11–15 post-inoculation, intermediate-stage: day 17–21 post-inoculation, late-stage: day 24–32 post-inoculation) as an additional validation towards broader application of the organ-organ analysis pipeline for TSPO-PET. In a head-to-head comparison, highly similar TSPO-PET patterns of glioblastoma-bearing mice were observed when using the tracers [^18^F]DPA-714 and [^18^F]GE-180 (Supplementary Fig. S3a). Late-stage glioblastoma-bearing mice showed no significant difference in median ICC (*p* = 0.061) as measured with [^18^F]DPA-714 (median ICC: 0.28 (0.03, 0.49)) compared to [^18^F]GE-180 (median ICC: 0.14 (-0.03, 0.51)) (Supplementary Fig. S3b). Furthermore, ICCs of late-stage glioblastoma-bearing mice scanned with [^18^F]DPA-714 showed a significant positive linear association with ICCs of those scanned with [^18^F]GE-180 (*β* = 0.62, *r* = 0.58, *p* < 10^− 4^) (Supplementary Fig. S3c).

**Fig. 4:**
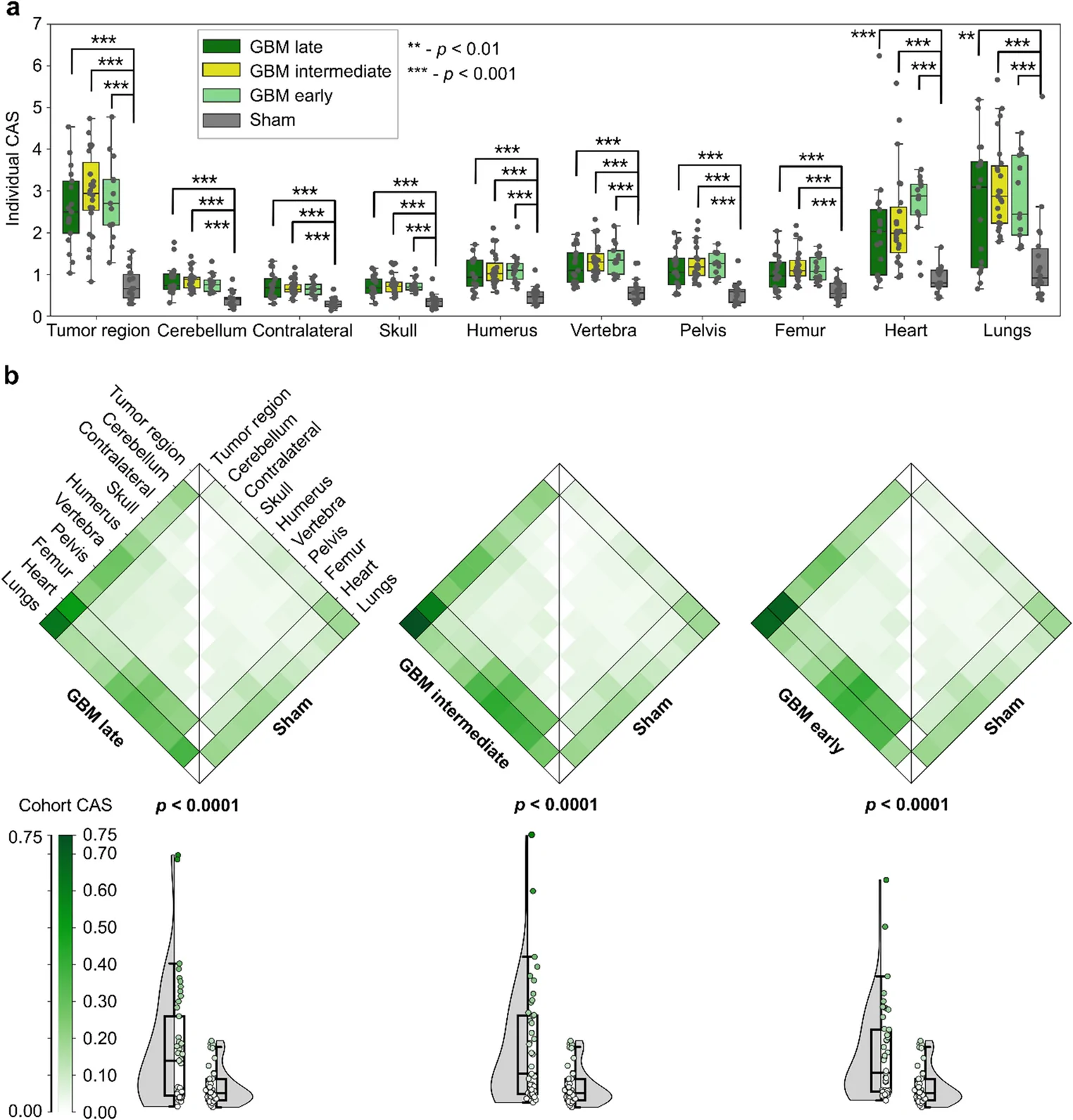
Assessment of correlation abnormality scores (CAS) showing the deviation of organ-organ correlations at the individual and cohort level of late-stage (GBM late), intermediate-stage (GBM intermediate), and early-stage glioblastoma-bearing mice (GBM early) in comparison with sham-injected mice (sham) using [^18^F]DPA-714 TSPO-PET signals. **a** Individual CAS for all volumes of interest (VOIs) and **b** cohort CAS of all VOI pairs

In the cohort with prolonged survival, a significant difference in median individual CAS was observed in all regions between sham-injected mice and glioblastoma-bearing mice (*p* < 0.01), already at the early stage (Fig. 4a). Similar to the results shown in Fig. 3a, the median individual CAS values in the heart (0.80), lungs (0.92), and tumor (0.66) of the [^18^F]DPA-714 TSPO-PET sham cohort were higher in comparison to those of the other regions of the sham cohort (< 0.59), further confirming greater variability compared to other regions (Fig. 4a). In the early-, intermediate-, and late-stage of glioblastoma-bearing mice, the median individual CASs of the heart (early: 2.88, intermediate: 1.99, late: 2.03), lungs (early: 2.45, intermediate: 2.87, late: 3.09), and tumor (early: 2.70, intermediate: 2.94, late: 2.50), increased and reached the highest values compared to that in the other regions within the glioblastoma cohorts (Fig. 4a).

The median cohort CAS increased significantly in all three glioblastoma stages compared to sham (late: 0.11 (0.06, 0.22), intermediate: 0.10 (0.05, 0.26), early: 0.14 (0.05, 0.26), sham: 0.05 (0.03, 0.09), *p* < 10^− 9^) (Fig. 4b). This may be due to the longer period between inoculation and the early-stage PET scan. Overall, there was a large similarity of changes in organ-organ interaction between the glioblastoma cohort measured with [^18^F]GE-180 (Fig. 3) and the glioblastoma cohort measured with [^18^F]DPA-714 (Fig. 4), supporting the robustness of the methodology.

**Fig. 5:**
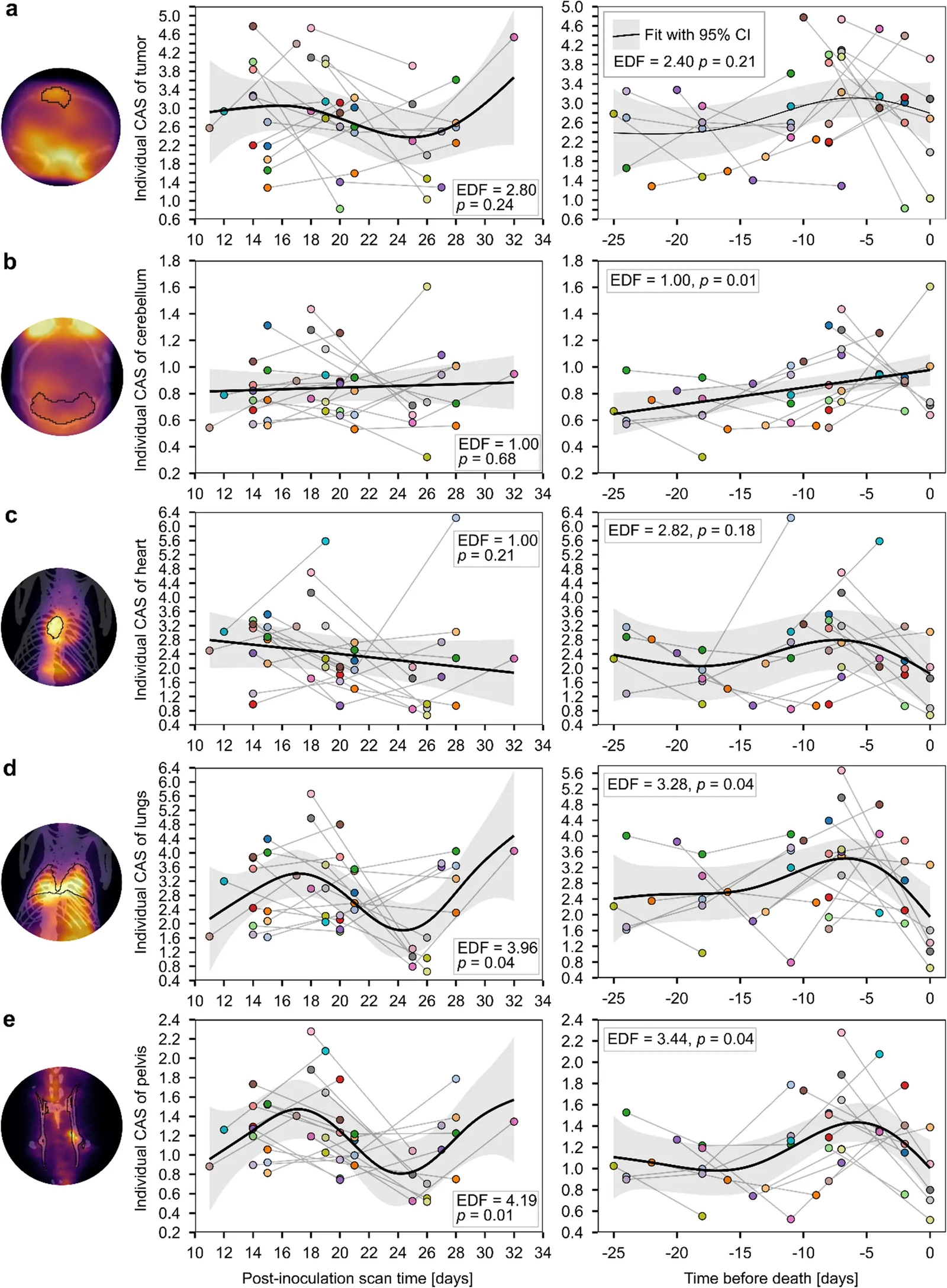
Longitudinal trend in the individual correlation abnormality scores (CAS) for various volumes of interest (VOIs) in glioblastoma-bearing mice using [^18^F]DPA-714 TSPO-PET. The longitudinal course of the individual CAS in **a** the tumor, **b** the cerebellum, **c** the heart, **d** the lungs, and **e** the pelvis of glioblastoma-bearing mice, modeled by the generalized additive mixed model (GAMM). Each color represents a single mouse. Grey lines connecting points of the same color represent repeated measurements from the same mouse

Finally, we applied a generalized additive mixed model to test for associations between longitudinal changes in TSPO expression and tumor progression. We observed distinct temporal associations between the individual CAS values and the time of the PET scan and the time before death (Fig. 5a-e). However, these associations were not apparent between TSPO-PET signal, particularly in the cerebellum and bones, and the time of the PET scan and the time before death (Supplementary Fig. S4). The model demonstrated that CAS values show two maxima at early- and late-stage, particularly in tumors (EDF = 2.80, *p =* 0.24) (Fig. 5a), lungs (EDF = 3.96, *p =* 0.04) (Fig. 5d), and bone (EDF = 4.19, *p =* 0.01) (Fig. 5e). Approximately one week before the death of the mice, the individual CAS in tumors (EDF = 2.40, *p* = 0.21), lungs (EDF = 3.82, *p* = 0.04), and bone (EDF = 3.44, *p* = 0.04) reach their maxima. The pelvis was used as a representative bone, while equal effects were seen for all other bones (EDF = 2.78–4.75, *p* = 0.004–0.16) (Supplementary Fig. S5). Contrary to most other individual CAS values, the individual CAS in the cerebellum demonstrated a continuous increase towards time of death (EDF = 1.00, *p* = 0.01) (Fig. 5b).

## Discussion

We investigated correlations of TSPO expression between organ systems at various stages of glioblastoma growth, while a detailed biological investigation of immune cross-talk was beyond the scope of this study. Pairwise Pearson correlation analysis, similar to previous attempts [1, 15, 22, 23], and CAS revealed stronger alterations in inter-organ correlations of TSPO-PET signals than single-organ TSPO-PET signals in glioblastoma-bearing mice compared to sham-injected controls. Altered inter-organ correlations of TSPO-PET signals were mainly driven by decreasing lung TSPO-PET signals. At early tumor stages, significant CAS changes occurred primarily in the tumor, whereas at late stages, these effects also became apparent in the heart and lungs. Higher sensitivity of CAS compared to single-organ TSPO-PET signal was reproduced with two different TSPO tracers ([^18^F]GE-180 and [^18^F]DPA-714) across different tumor stages.

Whole-body TSPO-PET has also been applied in cardiovascular research, with myocardial infarction as the investigated disease and neuroinflammation as the biological process of interest [8, 24]. Macrophage-depleted mice showed reduced myocardial TSPO-PET signal and CD68+ macrophage counts after myocardial infarction [24], supporting TSPO specificity for myeloid cells in the cardiac compartment.

Several studies have explored systemic immune cell phenotype changes in the presence of brain cancer, mostly to establish liquid biopsies for therapy monitoring [25]. Systemic immunosuppression via non-steroid soluble mediators in GL261 and CT2A glioblastoma-bearing mice [26], particularly showing reduced MHCII expression, aligns with our findings of reduced TSPO-PET signal in lungs and peripheral bones. Therefore, we hypothesize a redistribution of myeloid cells across bone compartments already at early tumor stages.

Importantly, PET imaging alone cannot distinguish whether reduced TSPO-PET signal and altered organ-organ interactions result from changes in TSPO expression or cellular abundance. If peripheral changes reflect immune cell migration toward the brain [27], the peripheral immune compartment could become a potential therapeutic target. We therefore hypothesize that targeting only the tumor microenvironment (TME) may be insufficient if dysfunctional peripheral immune cells continuously replenish the brain. If peripheral alterations instead reflect a phenotypic shift, they carry diagnostic and prognostic potential, enabling, e.g., non-invasive central nervous system immune monitoring, therapy response prediction, or early tumor recurrence detection [28]. In either case, our data support the concept that glioblastoma is not solely a brain disease and indicate an immune cross-talk between the brain and peripheral organs.

More specific PET targets, such as CD45 [29] and triggering receptor on myeloid cells 2 (TREM2) [30], could facilitate more precise immune system assessment. Whole-body TSPO-PET with immune cell tracers could be used for cross-correlation with non-invasive blood-derived immune cell signatures [31] in the presence of glioblastoma. In addition, whole-body PET analyses could also further our understanding of peripheral effects of local immune modulation, including the release of non-steroid soluble mediators [26]. Understanding the mechanistic pathways underlying brain-periphery immune communication could identify targets for future immunomodulatory interventions.

Unlike peripheral bones, skull bone marrow showed a relative increase in TSPO-PET signal. Contralateral skull segmentation served as an internal control to exclude partial-volume or spillover effects, providing a robust pipeline for future studies. This finding supports the active role of skull bone marrow in the brain–immune axis [32] and reflects inflammatory responses within the brain [33]. The skull bone marrow displays a distinct molecular profile [33], shaped by its anatomical proximity to the meninges, a key interface between peripheral and central nervous systems [33]. Furthermore, calvarial bone marrow has been identified as a source of antitumor myeloid antigen-presenting cells, including tumor-associated neutrophils that promote T-cell cytotoxicity and memory formation in glioblastoma [34]. In this context, future studies using TSPO tracers may enable labeling of hybrid inducible immature neutrophils originating from local skull bone marrow rather than the circulation in mouse glioblastoma models [34]. The absence of a comparable TSPO-PET signal in the skull in sham mice supports the specificity of the observed pattern to the glioblastoma model, despite potential confounding by blood-brain barrier permeability or peripheral inflammation [35].

The present study has some limitations. VOI segmentation was performed manually or semi-automatically, while a fully automated segmentation should be pursued to reduce computation time. TSPO is expressed not only by glial cells but also by glioma cells, myeloid cells within the TME [36], immune cells such as microglia and astrocytes [2, 36, 37], vascular endothelial cells, smooth muscle cells [38] and mouse macrophages [37], with the Human Protein Atlas [39] indicating predominant immune cell expression. The detailed cellular sources of TSPO-PET in different organ systems remain unclear, warranting multi-organ cell sorting after tracer injection in future studies. Additionally, modified glioma cells may affect immune infiltration [40], and normal cardiac metabolic perturbation may influence the myocardial TSPO-PET signal. The inclusion of the tracer [^18^F]DPA-714 was intended to reinforce the validity and enable longitudinal assessment; however, comparability with the [^18^F]GE-180 group is limited by differences in injected cell numbers, survival time, and scan timing. Furthermore, partial degradation of [¹⁸F]DPA-714 may contribute to increased calvarial bone uptake [41].

## Conclusions

Whole-body TSPO-PET enables the assessment of alterations in organ TSPO expression during glioblastoma progression. An analysis of whole-body TSPO connectivity revealed stage-dependent changes in TSPO expression in organs relevant to the immune system. Since diseases rarely affect only a single organ, it is necessary to assess changes throughout the entire body, particularly with regard to therapies. Our framework demonstrated that different organs can be affected in different ways, a fact that must be considered when diagnosing, predicting the prognosis of, and treating glioblastomas.

## Supplementary Information

Below is the link to the electronic supplementary material.

Supplementary Material (DOCX 2.61 MB)

## Data Availability

The datasets generated during and/or analysed during the current study are available from the corresponding author on reasonable request.
