# Supplementary material for: Whole-body TSPO-PET reveals stage-dependent disruption of systemic immune organ interactions in glioblastoma
Source: Eur J Nucl Med Mol Imaging. 2026 Jul 14;53(12):7059–70. doi: 10.1007/s00259-026-08057-0 (PMC13633316; doi:10.1007/s00259-026-08057-0)
Supplement: Supplementary file 1 [file 259_2026_8057_MOESM1_ESM.docx]

# Supplementary Materials

## Methods

### Animals

All animal experiments followed an established standardized protocol [1] and were performed according to the National Guidelines for Animal Protection, Germany, with the approval of the regional animal committee (Regierung von Oberbayern) and under the supervision of a veterinarian. Briefly, the animals were housed in an environment that was temperature- and humidity-controlled, and they were exposed to a 12-hour light-dark cycle. The animals had free access to food (Ssniff, Soest, Germany) and water.

Female, 6–8-week-old C57BL/6 mice were randomly divided into glioblastoma and sham-injected mice and scanned with [^18^F]GE-180 (*n* = 67) or [^18^F]DPA-714 (*n* = 39).

Within the [^18^F]GE-180 group, 49 mice were subject to stereotactic inoculation with 100,000 SB28-eGFP cells suspended in 2 μL of Dulbecco's modified Eagle's medium (DMEM) (Merck, Darmstadt, Germany) (glioblastoma-bearing mice), and 18 mice were inoculated with 2 μL of saline (sham-injected mice).

Within the [^18^F]DPA-714 group, 27 mice were subject to stereotactic inoculation with 1,000 SB28-eGFP cells suspended in 2 μL of Hanks Balanced Salt Solution (HBSS) (Thermo Fisher Scientific (Waltham, MA, USA)) (glioblastoma-bearing mice), and 12 mice were inoculated with 2 μL of HBSS (sham-injected mice).

Mice were anesthetized with intraperitoneal injections of 10% ketamine (100 mg/kg) and 2% xylazine (10 mg/kg) in 0.9% NaCl prior to inoculation. Under anesthesia, mice were immobilized and carefully mounted onto a stereotactic head holder (David Kopf Instruments, Tujunga, CA, USA) in the flat-skull position. Following a thorough surface disinfection, the skin of the skull was dissected using a scalpel blade. The skull was then meticulously drilled using a high-speed micromotor drill (Stoelting Co., Wood Dale, IL, USA) at a depth of 2 mm posterior and 1 mm left of the bregma.

Subsequently, a stereotactic injection technique was employed, whereby the cells were meticulously deposited into the target region with a Hamilton syringe (Hamilton, Bonaduz, Switzerland), employing a 10 μl Hamilton syringe. The injection was executed at a depth of 2 mm below the drill hole. The injection of cells was executed at a rate of 1×10^5^ or 1×10^3^ cells per minute, and the needle was then left in place for an additional 2 min to allow for the desired level of cell infiltration. Following this initial period, the needle was gradually withdrawn at a rate of 1 mm per minute. After this procedure, the wound was closed by suturing.

Mice were examined daily for tumor-related symptoms and were euthanized when the tumor burden (defined as 15% weight loss and/or the presence of other clinical symptoms, including appearance, coordinative deficits, and motor symptoms) reached stop criteria (not reached in any animal).

### Cell culture

The cell culture was prepared according to the established protocol [1]. SB28 cells do not express detectable CD40, representing a weakly immunogenic glioma model [2]. Briefly, SB28-GFP tumor cells were cultured in DMEM containing 4.5 g/L glucose, MEM non-essential amino acids (1x), 1% penicillin–streptomycin solution (Thermo Fisher Scientific, Waltham, MA, USA), and 10% fetal bovine serum (FBS, Biochrome, Berlin, Germany). The cell cultures were maintained at the Department of Nuclear Medicine at LMU Hospital in an incubator at 37 °C in a humidified atmosphere with 5% CO₂. The cells were passaged when the cell density in the flask reached 80% confluence. For passaging, the cells were detached using 1 mL of accutase (Sigma-Aldrich, Darmstadt, Germany).

### Small-animal PET/CT imaging

All small-animal PET experiments were performed under isoflurane anesthesia (1.5% at the time of tracer injection and during imaging; delivery: 3.5 liters/min).

A contrast-enhanced computed tomography (CT) scan was performed before the [^18^F]GE-180 PET scan for both the glioblastoma and the sham-injected mice. The contrast medium (0.3 mL of Iomeprol 300 M) was injected five minutes before the CT scan. A CT scan without contrast medium was performed before the [^18^F]DPA-714 PET scan for both the glioblastoma and the sham-injected mice.

PET reconstruction was performed using an Ordered Subsets Expectation Maximization (OSEM-3D) algorithm (4 iterations, 12 subsets) with decay correction, scatter correction, CT-based attenuation correction, dead time correction, and sensitivity normalization.

The resulting PET images had a matrix size of 225×225×235 voxels measuring 0.4×0.4×0.4 mm³. The data were binned into a total of 28 frames consisting of 6×10 s, 6×30 s, 6×60 s, and 10×300 s. The average of frames 25 to 28 (40 to 60 min) was used for analysis. The resulting CT images consisted of 430×430×443 voxels, each measuring 0.23×0.23×0.23 mm³.

### Radiosynthesis

Automated radiotracer production of [^18^F]GE-180 was performed using a FASTlab^TM^ synthesizer (GE Healthcare) with single-use disposable cassettes according to an established protocol [1]. Briefly, the pre-filled precursor vial was assembled on the cassette before the cassette was mounted on the synthesizer. The FASTlab^TM^ control software prompts were followed to run the cassette test and to start the synthesis. No carrier added, ^18^F-fluoride was produced via ^18^O(p, n)^18^F reaction by proton irradiation of ^18^O-enriched water and delivered to the ^18^F incoming reservoir. The fully automated manufacturing process consists of the following steps: trapping of ^18^F-fluoride on a QMA cartridge, elution using Kryptofix® 222, potassium hydrogen carbonate, water and acetonitrile, azeotropic drying of ^18^F-fluoride at 120°C for 9 min, labeling of the precursor in MeCN at 100 °C for 6 min, dilution of the crude product with water, tC18 cartridge based purification by use of 20 mL 40% (v/v) Ethanol and 11.5 mL 35% (v/v) Ethanol, elution of the product with 3.5 mL 55% (v/v) Ethanol and final formulation with phosphate buffer. The radiochemical yield of *n*=16 recorded syntheses was 39 ± 7% (non-decay corrected), and the radiochemical purity was ≥ 98% at a synthesis time of 43 min.

The radiotracer [^18^F]DPA-714 was synthesized by radiofluorination of a tosylate-based precursor on a TRASIS AllinOne synthesis module (Ans, Belgium). For this purpose, cyclotron-derived [^18^F]fluoride was trapped on an ion exchange cartridge and eluted with triethylammonium carbonate in aqueous acetonitrile. After azeotropic drying with acetonitrile and addition of the precursor, the reaction mixture was heated for 10 min at 100 °C, quenched with the addition of an ethanol solution, and purified using preparative High-performance liquid chromatography (HPLC). The product was then transferred to a CLIO dispensing unit and filled in portions.

### Statistical Analysis

To investigate reproducibility and robustness under sample size imbalance, random subsampling of the glioblastoma cohorts ([^18^F]GE-180 early- and late-stage) without replacement (*n* = 18 mice per iteration, matching the sham reference size, 1000 iterations) was performed. Each cohort CAS per volume of interest (VOI) pair showed a coefficient of variation (CV, CV = standard deviation / mean) across 1000 iterations that was below 0.16 for all VOI pairs, demonstrating stable reproducibility (Fig. S2a). As the individual CAS reflects a single mouse's deviation from the bootstrapped reference fit, it is by definition independent of the composition of the test cohort. Sample size imbalance and subsampling analyses were therefore only performed at the cohort level using the cohort CAS.

To assess sensitivity to outliers, two leave-one-out (LOO) analyses were performed. To assess sensitivity to outliers of the glioblastoma cohorts ([^18^F]GE-180; early-stage: *n* = 20, late-stage: *n* = 29), each glioblastoma-bearing mouse was sequentially excluded from the respective cohort with the reference fit held constant, and the resulting cohort CAS was calculated. Each cohort CAS per VOI pair showed a CV across all iterations (early: *n* = 20, late: *n* = 29) that was below 0.06 for all VOI pairs, demonstrating stable reproducibility (Fig. S2b). To assess sensitivity to outliers of the reference cohort ([^18^F]GE-180; sham-injected: *n* = 18), each sham-injected mouse was sequentially excluded from the reference cohort, and the resulting cohort CAS per VOI pair and individual CAS per mouse and VOI for the glioblastoma-bearing mice were calculated. The CV across 18 iterations remained below 0.35 for all cohort CAS per VOI pair (Fig. S2c) and below 0.23 for all individual CAS values per mouse and VOI (Fig. S2d).

Linear regression was performed between the resulting ICCs of the late-stage glioblastoma cohorts ([^18^F]GE-180 vs [^18^F]DPA-714) (Fig. S3c). From this, the slope (*β*), correlation coefficient (*r*), and associated significance values (*p*) were determined. To ensure the robustness of the results, a permutation test (*n* = 1000) was performed to check whether the observed regression slope could be explained by chance, and bootstrapping (*n* = 1000) was used to estimate confidence intervals (95% CI) for *β* and *r*.

### Longitudinal progression

The generalized additive mixed models (GAMMs) included a penalized P-spline smooth term for scan time and time before death (maximum basis dimension *k* = 10 for each) to capture the nonlinear temporal effects, and a random intercept for each mouse to account for repeated measurements. Model parameters were estimated using restricted maximum likelihood (REML) in the *mgcv* R package.

## Tables

**Table S1** Overview of statistical tests and models used for comparing tracer uptake in volume of interest (VOI), correlations, correlation abnormality scores (CAS), and longitudinal tumor progression

| **Analysis type** | **Test / Model** | **Comparison units** | **Family size** |
| --- | --- | --- | --- |
| **[^18^F]GE-180 uptake** | | | |
| Comparison of [^18^F]GE-180 uptake per VOI between cohorts (Fig. 1b-d) | Mann-Whitney U,  BH-FDR | All VOIs (VOI-to-VOI ratios) × all cohort pairs | *N*_VOIs_× *N*_cohort pairs_ = 12 × 3 = 36 |
| **Interregional correlation coefficient (ICC)** | | | |
| Correlation between VOIs per cohort | Pearson’s r, BH-FDR | All pairwise VOI combinations per cohort | All directed pairs: *N*_VOIs_ × (*N*_VOIs_−1) = 90; *N*_VOIs_ = 10 |
| Comparison of median ICC ([^18^F]GE-180) between GBM (early, late) and sham (Fig. 2) | Wilcoxon signed-rank (two-tailed) | VOI-pair-matched (*N* = 45), per cohort comparison | *N*_cohort pairs_ = 2 |
| **Correlation abnormality score (CAS)** | | | |
| Comparison of median individual CAS ([^18^F]GE-180) per VOI between cohorts (Fig. 3a) | Mann-Whitney U,  BH-FDR | All VOIs × all cohort pairs | *N*_VOIs_ × *N*_cohort pairs_ = 10 × 3 = 30 |
| Comparison of median cohort CAS ([^18^F]GE-180) between GBM and sham (Fig. 3b) | Wilcoxon signed-rank (two-tailed) | VOI-pair-matched, per cohort comparison | *N*_cohort pairs_ = 2 |
| Comparison of median individual CAS ([^18^F]DPA-714 ) per VOI between cohorts (Fig. 4a) | Mann-Whitney U,  BH-FDR | All VOIs × all cohort pairs | *N*_VOIs_ × *N*_cohort pairs_ = 10 × 4 = 40 |
| Comparison of median cohort CAS ([^18^F]DPA-714) between GBM and sham (Fig. 4b) | Wilcoxon signed-rank (two-tailed) | VOI-pair-matched, per cohort comparison | *N*_cohort pairs_ = 3 |
| **Longitudinal GAMM analysis (*n* = 19 mice)** | | | |
| Temporal trajectory of individual CASs vs post-inoculation scan time per VOI (Fig. 5, Fig. S5) | GAMM - penalized P-spline smooth (*k* = 10) + random intercept per mouse | Single smooth term per VOI | *N*_VOIs_ = 10 |
| Temporal trajectory of individual CAS vs time before death per VOI (Fig. 5, Fig. S5) | GAMM - penalized P-spline smooth (*k* = 10) + random intercept per mouse | Single smooth term per VOI | *N*_VOIs_ = 10 |
| **Supplementary materials** | | | |
| **[^18^F]GE-180 vs [^18^F]DPA-714 late-stage comparisons** | | | |
| Comparison of median ICC between [^18^F]GE-180 and [^18^F]DPA-714 late-stage cohorts (Fig. S3b) | Wilcoxon signed-rank (two-tailed) | VOI-pair-matched, per cohort comparison | *N*_cohort pairs_ = 1 |
| ICC correlation between [^18^F]GE-180 and [^18^F]DPA-714 late-stage cohorts across VOI pairs (Fig. S3c) | Linear regression | Single regression across all VOI-pairs (*N*_VOI pairs_ = 45) | *N*_cohort pairs_ = 1 |
| **Longitudinal GAMM analysis (*N* = 19 mice)** | | | |
| Temporal trajectory of [^18^F]DPA-714 uptake vs post-inoculation scan time per VOI (Fig. S4) | GAMM - penalized P-spline smooth (*k* = 10) + random intercept per mouse | Single smooth term per VOI | *N*_VOIs_ = 10 |
| Temporal trajectory of [^18^F]DPA-714 uptake vs time before death per VOI (Fig. S4) | GAMM - penalized P-spline smooth (*k* = 10) + random intercept per mouse | Single smooth term per VOI | *N*_VOIs_ = 10 |

## Figures

**Fig. S1** Schematic overview of mouse cohorts and performed PET/CT scans. *n* = 67 mice underwent PET/CT scans using the tracer [^18^F]GE-180 (top) and *n* = 39 mice using the tracer [^18^F]DPA-714 (bottom). The [^18^F]GE-180 mice were divided into *n* = 49 glioblastoma-bearing (GBM) mice and *n* = 18 sham-injected (sham) mice, with the GBM mice being inoculated with 100,000 tumour cells and the sham mice with saline. Furthermore, the [^18^F]GE-180 GBM mice were divided into an early-stage (*n* = 20, scan time: day 5–13) and a late-stage (*n* = 29, scan time: day 18–19) group. The [^18^F]DPA-714 mice were divided into GBM mice (*n* = 27) and sham mice (*n* = 12), with the GBM mice being inoculated with 1,000 tumour cells and the sham mice with HBSS. [^18^F]DPA-714 mice were scanned 1–3 times, resulting in *n* = 18 scans for sham mice and *n* = 53 scans for GBM mice. The *n* = 53 scans of the GBM mice were further divided into early-stage (*n* = 13, scan time: day 11–15), intermediate (*n* = 23, scan time: day 17–21), and late-stage (*n* = 17, scan time: day 24–32) groups

**Fig. S2** Reproducibility, sensitivity, and robustness of the individual and cohort correlation abnormality score (CAS). **a** Random subsampling of the glioblastoma cohorts (GBM late, GBM early) without replacement (n = 18 mice per iteration, matching the sham reference size, 1000 iterations). **b** Leave-one-out analysis (LOO) for assessing sensitivity to outliers of the glioblastoma cohorts: each glioblastoma-bearing mouse was sequentially excluded from the respective cohort (early-stage: n = 20, late-stage: n = 29) with the reference fit held constant, and the resulting cohort CAS was calculated. **c** Leave-one-out analysis for assessing sensitivity to outliers of the sham cohort (LOO-Sham): each sham mouse was sequentially excluded from the reference cohort, and the resulting cohort CAS per volume of interest (VOI) pair was recalculated (n = 18 iterations). The coefficient of variation (CV) of the cohort CAS remained below 0.35 for all VOI pairs. **d** Leave-one-out analysis for assessing sensitivity to outliers of the sham cohort (LOO-Sham): each sham mouse was sequentially excluded from the reference cohort, and the resulting individual CAS per mouse and VOI was recalculated (n = 18 iterations). The CV remained below 0.23 for all individual CAS values per mouse and VOI

**Fig. S3** Comparison of [^18^F]GE-180 TSPO-PET and [^18^F]DPA-714 TSPO-PET. **a** Head-to-head comparison of [^18^F]GE-180 and [^18^F]DPA-714 showing a glioblastoma-bearing mouse scanned with [^18^F]GE-180 at day 8 post-inoculation (top) and with [^18^F]DPA-714 at day 9 post-inoculation (bottom). **b** The distribution of interregional correlation coefficients (ICCs) shows no significant difference between late-stage glioblastoma-bearing mice (GBM late) measured with [^18^F]GE-180 and [^18^F]DPA-714. p-values derived from the Wilcoxon signed-rank test. **c** ICCs of late-stage glioblastoma-bearing mice (GBM late) scanned with [^18^F]DPA-714 showed a significant positive linear association with ICCs of those scanned with [^18^F]GE-180. *β*-, *r*-, and *p*-value derived from the linear regression model

**Fig. S4** Longitudinal trend in the mean [^18^F]DPA-714 uptake ratio of various volumes of interest (VOIs), normalized to the whole body in glioblastoma-bearing mice, using [^18^F]DPA-714 TSPO-PET. The longitudinal course of the mean [^18^F]DPA-714 uptake ratio of **a** the tumor, **b** the cerebellum, **c** the heart, **d** the lungs, and **e** the pelvis, normalized to the whole body of glioblastoma-bearing mice, was modeled by the generalized additive mixed model (GAMM). The significance of the smooth terms that capture nonlinear temporal effects was assessed using p-values and interpreted alongside effective degrees of freedom (EDF). The data are shown with a nonlinear fit and the 95% confidence interval (CI). Each color represents a single mouse. Grey lines connecting points of the same color represent repeated measurements from the same mouse

**Fig. S5** Longitudinal trend in the individual correlation abnormality scores (CAS) of various bones in glioblastoma-bearing mice using [^18^F]DPA-714 TSPO-PET. The longitudinal course of the individual correlation abnormality scores (CAS) in the **a** the skull, **b** the humerus**, c** the vertebra, and **d** the femur of glioblastoma-bearing mice was modeled by the generalized additive mixed model (GAMM). The significance of the smooth terms that capture nonlinear temporal effects was assessed using p-values and interpreted alongside effective degrees of freedom (EDF). The data are shown with a nonlinear fit and the 95% confidence interval (CI). Each color represents a single mouse. Grey lines connecting points of the same color represent repeated measurements from the same mouse

## References

1.	Bartos LM, Kirchleitner SV, Blobner J, Wind K, Kunze LH, Holzgreve A, et al. 18 kDa translocator protein positron emission tomography facilitates early and robust tumor detection in the immunocompetent SB28 glioblastoma mouse model. Front Med (Lausanne). 2022;9:992993. doi:10.3389/fmed.2022.992993.

2.	Genoud V, Marinari E, Nikolaev SI, Castle JC, Bukur V, Dietrich PY, et al. Responsiveness to anti-PD-1 and anti-CTLA-4 immune checkpoint blockade in SB28 and GL261 mouse glioma models. Oncoimmunology. 2018;7:e1501137. doi:10.1080/2162402X.2018.1501137.
